# Impact of ribonucleotide incorporation by DNA polymerases β and λ on oxidative base excision repair

**DOI:** 10.1038/ncomms10805

**Published:** 2016-02-26

**Authors:** Emmanuele Crespan, Antonia Furrer, Marcel Rösinger, Federica Bertoletti, Elisa Mentegari, Giulia Chiapparini, Ralph Imhof, Nathalie Ziegler, Shana J. Sturla, Ulrich Hübscher, Barbara van Loon, Giovanni Maga

**Affiliations:** 1 DNA Enzymology & Molecular Virology Unit, Institute of Molecular Genetics IGM-CNR, via Abbiategrasso 207, I-27100 Pavia, Italy; 2 Department of Molecular Mechanisms of Disease, University of Zürich, CH-8057 Zürich, Switzerland; 3 Department of Health Sciences and Technology, ETH Zurich, CH-8092 Zürich, Switzerland

## Abstract

Oxidative stress is a very frequent source of DNA damage. Many cellular DNA polymerases (Pols) can incorporate ribonucleotides (rNMPs) during DNA synthesis. However, whether oxidative stress-triggered DNA repair synthesis contributes to genomic rNMPs incorporation is so far not fully understood. Human specialized Pols β and λ are the important enzymes involved in the oxidative stress tolerance, acting both in base excision repair and in translesion synthesis past the very frequent oxidative lesion 7,8-dihydro-8-oxoguanine (8-oxo-G). We found that Pol β, to a greater extent than Pol λ can incorporate rNMPs opposite normal bases or 8-oxo-G, and with a different fidelity. Further, the incorporation of rNMPs opposite 8-oxo-G delays repair by DNA glycosylases. Studies in Pol β- and λ-deficient cell extracts suggest that Pol β levels can greatly affect rNMP incorporation opposite oxidative DNA lesions.

## Abstract (web summary)

Oxidative stress is a common source of DNA damage and is repaired by the base excision repair machinery, including polymerase beta. Here the authors find that polymerase beta, and to a lesser extent lambda, can mistakenly incorporate ribonucleotides during synthesis.

## Graphical abstract

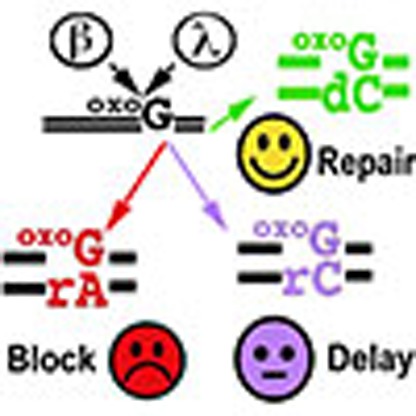

DNA polymerases (Pols) are specialized enzymes that selectively use deoxynucleoside triphosphates (dNTPs) as building blocks for DNA synthesis. However, despite their specificity, replicative Pols can incorporate ribonucleoside monophosphates (rNMPs) during DNA replication (more than 1,000,000 rNMPs per mammalian genome)^1^,^2^,^3^. Such rNMPs incorporation has been suggested to assist recognition of the newly synthesized strand by mismatch repair^4^,^5^. However, it can also constitute a major threat to genome stability^6^,^7^. The sugar-phosphate backbone of RNA is much more prone to strand breakage than DNA, potentially resulting in the accumulation of strand breaks. In addition, rNMPs incorporation could decrease the rate of DNA replication^2^,^8^,^9^,^10^. Due to the intrinsic 3′→5′ proofreading exonuclease activity replicative Pols can excise incorporated rNMPs to a variable extent^9^,^11^. The majority of rNMPs is however removed by the RNaseH2-dependent ribonucleotide excision repair^12^. Genetic studies in yeast have shown that RNaseH2 mutants suffer replication stress and require translesion synthesis (TLS) to avoid accumulation of rNMPs in the genome^13^. The embryonic lethality of RNaseH2-null mice as well as the severe phenotype of the Aicardi–Goutières syndrome in humans, due to mutations in RNaseH2, indicate that removal of rNMPs from the DNA is essential also in mammals^14^.

Besides replication, DNA repair is another potential source of rNMPs incorporation. This may become particularly relevant in post-mitotic cells, such as neurons. To date, 17 Pols, including the cytidyl-transferase Rev1 and telomerase, have been identified in human cells^15^, many of which are specialized in various DNA repair pathways. Among them, family X enzymes Pols β, μ and λ participate in base excision repair (BER), non-homologous end joining and in specialized form of TLS of oxidative lesions^16^,^17^. Several studies have shown that family X Pols can incorporate rNMPs during synthesis of undamaged DNA (reviewed in ref. 18), but with varying sugar selectivity. Pol μ displayed the lowest discrimination, in the range of 1- to 10-fold preference for dNMPs over rNMPs incorporation^19^,^20^. On the other hand, Pols β and λ showed sugar selectivity in the 3,000–50,000 range, depending on the particular base pair involved^21^,^22^,^23^. While Pol μ lacks a suitable ‘steric gate' amino acid side chain (usually Glu, Tyr or Phe), used by the majority of Pols for sugar discrimination^24^, Pols β and λ achieve the sugar selection using a protein backbone segment to exclude rNTPs^21^,^23^. Thus, both the active site architecture and the particular mechanism of sugar selection may account for the very diverse range of selectivity measured for the different Pols.

Genomes of living organisms are constantly exposed to various damaging agents. Among the most frequently generated DNA base modifications, are the oxidative lesions, particularly 7,8-dihydro-8-oxoguanine (8-oxo-G). During DNA replication 8-oxo-G lesions in the template strand lead to high frequency of misincorporation, generating A:8-oxo-G mismatches. The repair of these mismatches requires the sequential action of the MutY homologue (MutYH) and 8-oxo-G DNA glycosylase (OGG1), as well as of a specialized TLS Pol. We have previously shown that Pol λ is the most accurate in MutYH-initiated pathway, ensuring correct dCMP incorporation opposite 8-oxo-G, while Pol β can substitute for Pol λ, but at the expense of a reduced fidelity, leading to frequent misincorporation of dAMP opposite the lesion^25^. So far, it is not known whether DNA repair synthesis by Pols β and λ, either during BER or 8-oxo-G bypass, could contribute to genomic rNMPs accumulation. Since approximately 1,000–10,000 oxidative DNA lesions, of which 8-oxo-G is one of the most abundant, are generated spontaneously every day in each cell^26^, understanding to what extent repair Pols incorporate rNMPs and how this event could influence DNA repair, remains an important issue.

In the present study, we compare the ability of the two BER Pols β and λ to incorporate rNMPs versus dNMPs opposite all four undamaged bases as well as 8-oxo-G, within a range of Mg^2+^, Mn^2+^ and nucleotide concentrations matching those believed to normally occur in the cell. In addition, we examine the impact of rNMPs incorporation opposite 8-oxo-G on the subsequent BER initiated by the human OGG1 (hOGG1) and MutYH glycosylases. Finally, we measure incorporation of rNMPs opposite 8-oxo-G in extracts from cells deficient in either Pol λ or Pol β. Our results suggest that Pol β, to a greater extent than Pol λ, plays a role in rNMP incorporation opposite oxidative DNA damage.

## Results

### Metal-dependent rNMPs incorporation by Pol β and λ

**Figure 1:**
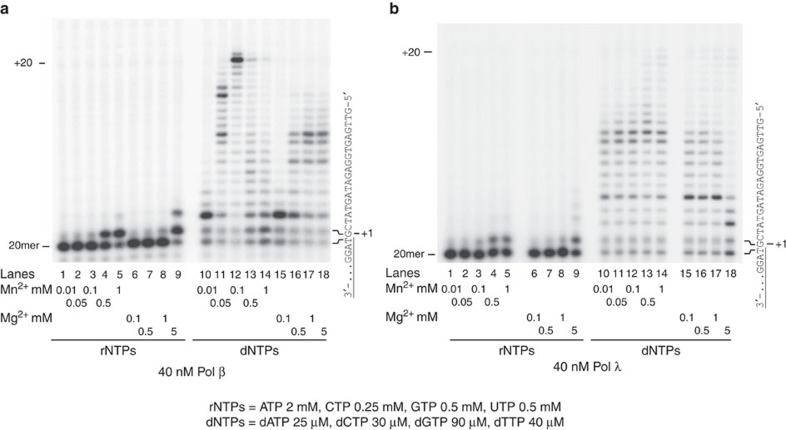
Nucleotide incorporation by DNA polymerases β and λ under different Mg^2+^ and Mn^2+^ conditions. (**a**,**b**) Pol β (**a**) and λ (**b**) activity was assayed in the presence of increasing Mn^2+^ or Mg^2+^ concentrations and with all four rNTPs or dNTPs at fixed concentrations in their physiological range. The sequence of the template strand of the 5′-labelled 20-G/40mer p/t is shown on the right side of each panel. The rNTPs and dNTPs concentrations used are indicated at the bottom of each panel.

The identity of the divalent metal cation Mg^2+^ or Mn^2+^, required by all Pols for catalytic activity^27^, can profoundly influence both Pol efficiency and the fidelity. The levels of Mg^2+^ and Mn^2+^ in the cells are tightly regulated and their physiological range of concentrations is quite different. While Mg^2+^ on average is present at concentrations ranging from 1 to 20 mM (refs 28, 29), Mn^2+^ is usually found in the 0.01–0.2 mM range^30^,^31^. Different studies have shown that Pols β (ref. 23) and λ (ref. 22) can incorporate rNMPs *in vitro*. However, these studies often were conducted in the presence of one metal activator (either Mg^2+^ or Mn^2+^) at a fixed dose. To compare the influence of Mg^2+^ and Mn^2+^ on the ability of Pols β and λ to incorporate rNMPs, DNA polymerization assays were performed on a 20/40mer primer/template (p/t) oligonucleotide, in the presence of different concentrations of the two metal activators, encompassing their respective physiological ranges. Similarly, mixtures of all four rNTPs or dNTPs were non-equimolar, to ensure that each individual nucleotide matched the previously published cellular concentrations^32^,^33^. As shown in Fig. 1a,b, Pols β and λ were able to incorporate rNMPs at physiological (1–5 mM) Mg^2+^ concentrations, while significant rNMPs incorporation in the presence of Mn^2+^ occurred only at 0.5–1 mM concentrations, higher than those normally measured in the cell. Thus, Mg^2+^ appeared to be the most relevant physiological activator for rNMPs incorporation by Pols β and λ, even though it cannot be excluded that in particular cell types or physiological conditions, Mn^2+^ concentrations may be high. Next, the fidelity of rNMPs incorporation was tested for all the 16 possible base pairs in the presence of Mg^2+^. It must be noted here that commercial rNTPs stocks often contain small (<1%) amounts of contaminating dNTPs, which, owing to the relatively high rNTPs concentrations used in the assay and the high affinity of Pols λ and β for the nucleotides, could also be incorporated. However, since the products resulting from rNMP incorporation were migrating slower than those arising from dNMP incorporation, they could be easily distinguished on the sequencing gels. As shown in Supplementary Fig. 1A, Pol λ was able to insert each rNMP opposite its complementary templating base (lanes 5, 9, 19 and 25). No significant misincorporation was observed, with the exception of very low rGMP incorporation opposite G (lane 17). Pol β (Supplementary Fig. 1B), on the other hand, was able to incorporate rCMP opposite G and, to a lower extent, rGMP opposite C (lanes 19 and 27), while rUMP and rAMP incorporation opposite their complementary bases was at the limit of detection (lanes 7 and 11).

Overall, these data revealed differences in the ability and fidelity of Pols β and λ to select and incorporate rNMPs opposite undamaged bases.

### rNMPs incorporation opposite 8-oxo-G by Pol λ

**Figure 2:**
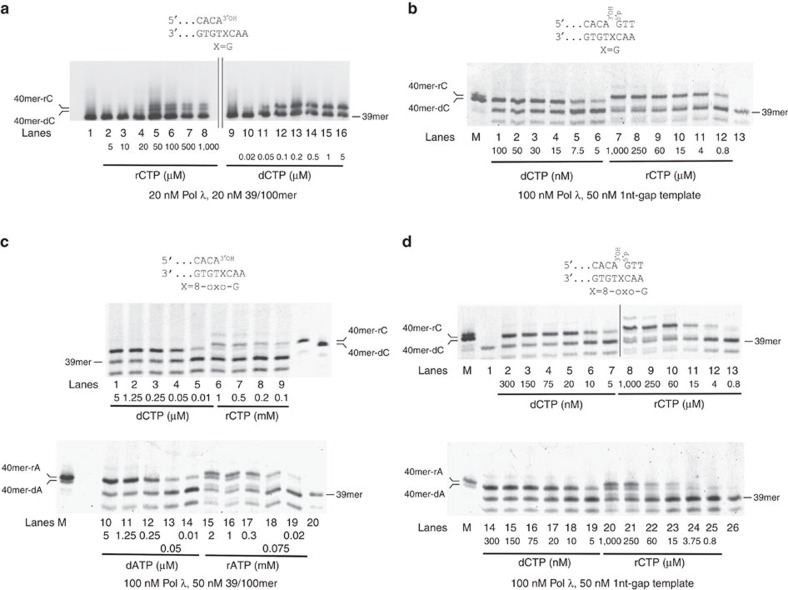
The fidelity of 8-oxo-G bypass by DNA polymerase λ in the presence of rNTPs is influenced by the structure of the DNA template. (**a**) Pol λ activity was measured on the undamaged 5′-labelled 39/100mer p/t, in the presence of increasing concentrations of rCTP or dCTP. Lane 1, control reaction in the absence of nucleotides. (**b**) As in **a**, but in the presence of the undamaged 5'-labelled 39/60/100mer 1 nt-gap template. M, a mixture of 5′-labelled 40mer oligonucleotides bearing either dCMP or rCMP as the terminal nucleotide, as markers. Lane 13, control reaction in the absence of nucleotides. (**c**) Pol λ activity was measured on the 5′-labelled 39/100mer p/t containing 8-oxo-G, in the presence of different concentrations of dCTP and rCTP (top panel), or dATP and rATP (bottom panel). 5′-Labelled 40mer oligonucleotides bearing either dCMP, rCMP, dAMP or rAMP as the terminal nucleotide, were used as markers. Lane 20, control reaction without nucleotides. (**d**) As in **c**, but in the presence of the 5'-labelled 39/60/100mer 1 nt-gap 8-oxo-G template. Lane M, 5′-labelled 40mer oligonucleotides bearing either dCMP, rCMP, dAMP or rAMP as the terminal nucleotide, were used as markers.

Besides undamaged DNA, repair Pols could potentially incorporate rNMPs opposite damaged DNA bases. 8-oxo-G, a frequent oxidative base damage, is a cognate substrate of Pol λ (refs 34, 35). To test the ability of Pol λ to use rNTPs for incorporation opposite 8-oxo-G, Pol λ activity was determined in the presence of each of the four rNTPs on a 39/100mer p/t or a single nucleotide (1 nt)-gap template, both bearing at the +1 position either 8-oxo-G, or G. The 1 nt-gap template was chosen since it resembles a BER intermediate. On both templates, Pol λ significantly incorporated only rCMP opposite a normal G (Supplementary Fig. 2A, lane 4; Supplementary Fig. 2C, lane 10), while it incorporated both rCMP and rAMP opposite and 8-oxo-G (Supplementary Fig. 2B,C, lanes 5 and 6). On both substrates, some incorporation opposite a normal G or 8-oxo-G was also occurring in the presence of rGTP (Supplementary Fig. 2A, lane 2; Supplementary Fig. 2B, lane 3; Supplementary Fig. 2C, lanes 3 and 8). However, as indicated by the faster electrophoretic mobility of the resulting +1 product, such events were due to contaminating dNTPs in the commercial rGTP stock. Only minimal rGMP incorporation was seen opposite 8-oxo-G on the 1 nt-gap template (Supplementary Fig. 2C, lane 3). Incorporation was monitored as a function of dCTP, rCTP, dATP or rATP concentrations (Fig. 2) on both substrates and the corresponding kinetic parameters and sugar selectivity are reported in Table 1. Pol λ showed a 7,500-fold selectivity for dCTP over rCTP as substrates for synthesis opposite a normal G on the BER-mimicking template, which was in line with previously published data^22^. However, this selectivity increased to 14,000 opposite an 8-oxo-G. On this template, Pol λ showed also a strong preference for dAMP incorporation versus rAMP opposite an 8-oxo-G, with a selectivity of 17,200. Interestingly, the relative fidelity of 8-oxo-G bypass by Pol λ on the BER-mimicking template was not changed in the presence of rNTPs, since rCTP was preferred over rATP as a substrate, to a similar extent as dCTP over dATP (5.5-fold and 4.5-fold, respectively). On the 39/100-mer p/t, Pol λ showed a 5,140-fold selectivity for dCMP versus rCMP incorporation opposite a normal G, which increased to 12,800 opposite an 8-oxo-G. Interestingly, both the selectivity and the fidelity of Pol λ for rAMP incorporation opposite 8-oxo-G, were decreased on this substrate, with respect to the 1 nt gap. The selectivity value for dAMP versus rAMP was 3,000-fold, while the preference for rCMP versus rAMP incorporation was 0.9, indicating that both rNMPs could be equally used by Pol λ, to bypass the lesion on this template.

Taken together, these data clearly indicated that the structure of the DNA substrate influenced the fidelity and sugar selectivity of Pol λ during 8-oxo-G bypass in the presence of rNTPs.

### Fidelity of rNMPs incorporation opposite 8-oxo-G by Pol β

**Figure 3:**
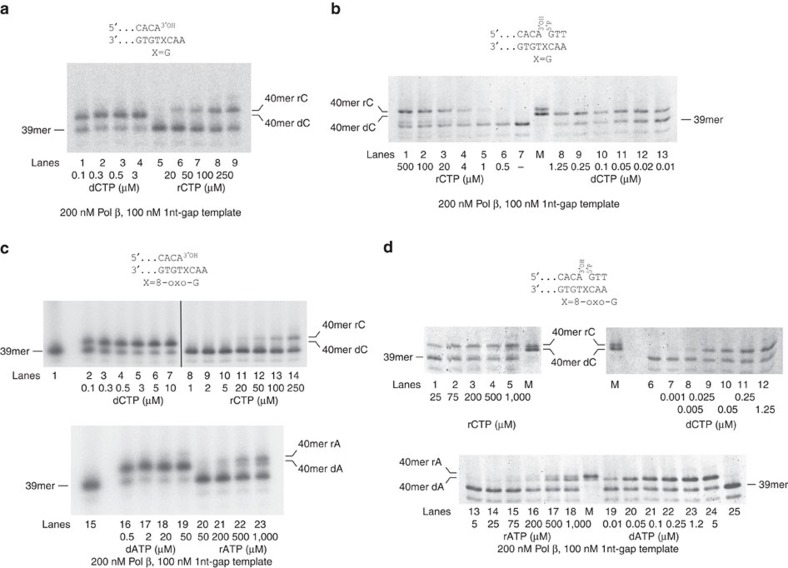
DNA polymerase β bypasses 8-oxo-G more faithfully in the presences of rNTPs than dNTPs. (**a**) Pol β activity was measured on the undamaged 5′-labelled 39/100mer p/t, in the presence of increasing concentrations of rCTP or dCTP. (**b**) As in **a**, but in the presence of the undamaged 5'-labelled 39/60/100mer 1 nt-gap template. M, a mixture of 5′-labelled 40mer oligonucleotides bearing either dCMP or rCMP as the terminal nucleotide, as markers. Lane 7, control reaction in the absence of nucleotides. (**c**) Pol β activity was measured on the 5′-labelled 39/100mer p/t containing 8-oxo-G, in the presence of different concentrations of dCTP and rCTP (top panel), or dATP and rATP (bottom panel). 5′-Labelled 40mer oligonucleotides bearing either dCMP, rCMP, dAMP or rAMP as the terminal nucleotide, were used as markers. Lanes 1 and 15, control reactions without nucleotides. (**d**) As in **c**, but in the presence of the 5'-labelled 39/60/100mer 1 nt-gap 8-oxo-G template. Lanes M, 5′-labelled 40mer oligonucleotides bearing either dCMP, rCMP, dAMP or rAMP as the terminal nucleotide, were used as markers. Lane 25, control reaction in the absence of nucleotides.

Pol β has been shown to frequently misincorporate dAMP opposite an 8-oxo-G^34^. As shown in Supplementary Fig. 2D, with the 1 nt gap BER-mimicking template, Pol β readily incorporated both rCMP and rAMP opposite 8-oxo-G (lanes 1 and 2), while only rCMP was inserted opposite a normal G (lane 9). As already noted above, with rUTP and rGTP, only incorporation of contaminating dNTPs was detected. Substrate titrations with increasing concentrations of dCTP, rCTP, dATP or rATP were performed on both the 39/100 mer p/t and the 1 nt-gap templates (Fig. 3). The results of the kinetic analysis (Table 1) showed that on the 1 nt gap BER-mimicking template Pol β displayed a sugar selectivity for dCTP versus rCTP as substrates for the incorporation opposite a normal G of 3,400, which is in agreement with published data^18^,^23^. Contrary to Pol λ, this value was not increased in the presence of an 8-oxo-G lesion and measured 3,200. Interestingly, on the same template containing 8-oxo-G, Pol β showed strong discrimination against rAMP incorporation, with selectivity value of 25,600. The preference for dAMP versus rAMP incorporation opposite 8-oxo-G was 13.4-fold, while the one for dCMP versus dAMP incorporation was only 1.65-fold. On the p/t, the selectivity values were 1,690 and 4,680 for dCMP versus rCMP incorporation opposite a normal G and an 8-oxo-G, respectively. On this template however, the selectivity for dAMP versus rAMP incorporation opposite the lesion was 2,800. The fidelity was similar, with a twofold preference for rCMP versus rAMP utilization and a threefold for dCMP versus dAMP.

These data suggested that Pol β was sensitive to the structure of the DNA template, similarly to Pol λ. In particular, while Pol β showed lower sugar selectivity for rCMP versus dCMP incorporation than Pol λ on the 1 nt gap BER-mimicking template, its bypass fidelity with rNMPs was higher, since it strongly preferred rCMP versus rAMP incorporation opposite the lesion.

To directly visualize simultaneous incorporation of rCMP or dCMP opposite 8-oxo-G, competition experiments were carried out on the 1 nt-gap template (Supplementary Fig. 2E). The products coming from the incorporation of either rCMP or dCMP could be distinguished on the gel, as they differ in electrophoretic mobility. By titrating increasing amounts of one nucleotide (either dCTP or rCTP), in the presence of a fixed amount of the other competing nucleotide, both Pol λ (lanes 1–11) and Pol β (lanes 12–16) showed a dose-dependent increase in the band corresponding to the nucleotide titrated into the reaction, while the band corresponding to the incorporation of the nucleotide at a fixed concentration, decreased. These experiments confirmed that rCTP and dCTP compete as substrates for incorporation opposite 8-oxo-G by both Pols.

### rNMPs opposite 8-oxo-G impair repair by hOGG1 and MutYH

**Figure 4:**
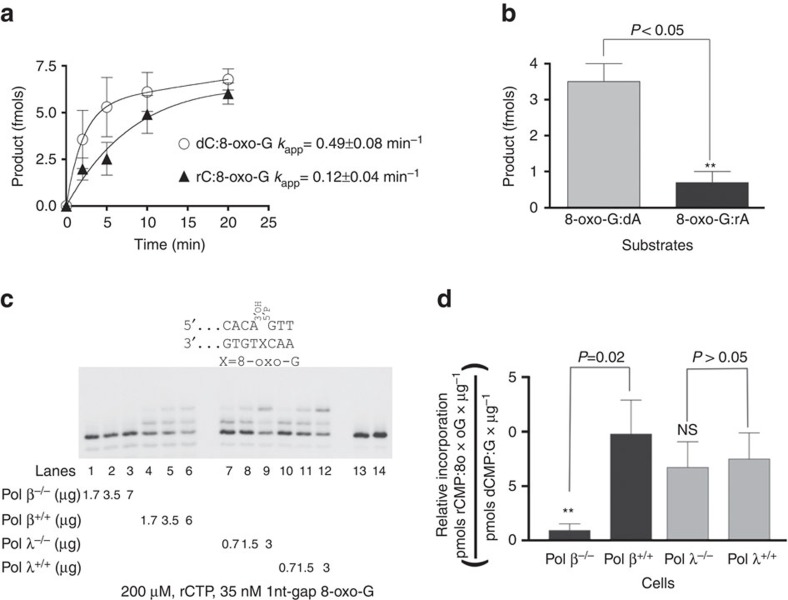
Incorporation of rNMPs opposite 8-oxo-G inhibits DNA repair and is reduced in the absence of DNA polymerase β. (**a**) Time course of the excision products accumulation generated by hOGG1 in the presence of a 8-oxo-G:dC (empty circles) or 8-oxo-G:rC (filled triangles) base pairs. The *k*_app_ values refer to the apparent rates for the exponential phase. Values are the mean of three independent replicates, error bars represent ±s.d. A representative experiment is shown in Supplementary Fig. 3A. (**b**) Quantification of the excision products generated by MutYH in the presence of a 8-oxo-G:dA (grey bars) or 8-oxo-G:rA (black bars) mismatch. Values are the mean of three independent replicates as the one presented in Supplementary Fig. 3B, error bars represent ±s.d. The *P* values were calculated by two-tailed Student's *t*-test. (**c**) Increasing amounts of the different cell extracts were titrated in the presence of the 1 nt-gap template bearing an 8-oxo-G and 200 μM rCTP. Lanes 13 and 14, control reactions in the absence of extracts. (**d**) The rCMP incorporation activity (expressed as pmols of rCTP incorporated opposite 8-oxo-G per μg of proteins of each extract) was normalized to the total DNA polymerase activity (expressed as pmols of dCMP incorporated opposite undamaged dG per μg of proteins of each extract). Values are the mean of three independent replicates like the one shown in **c**, error bars represent ±s.d. The *P* values were calculated by two-tailed Student's *t*-test.

Removal of 8-oxo-G paired to dC in double-stranded (ds) DNA, is predominantly accomplished by the BER glycosylase OGG1 (ref. 36). Since the data presented above clearly showed that rCMP could be incorporated opposite 8-oxo-G by the specialized repair Pols β and λ, we next asked whether this fundamental glycosylase reaction was affected by pairing of rC base to an 8-oxo-G. A glycosylase assay was performed, with hOGG1 and a ds DNA oligonucleotide bearing either a dC:8-oxo-G or a rC:8-oxo-G base pair. Interestingly, the 8-oxo-G incorporated opposite rCMP could be removed by hOGG1, even though at a fourfold slower rate (Fig. 4a; Supplementary Fig. 3A). Besides rC:8-oxo-G pair, both Pols β and λ may also generate rA:8-oxo-G mispairs (Figs 2 and 3). MutYH is the BER glycosylase responsible for the recognition and removal of dA erroneously paired with 8-oxo-G^37^. To address if MutYH can also act on rA:8-oxo-G mispair, a glycosylase assay was performed using ds DNA oligonucleotide substrate bearing either a dA:8-oxo-G or a rA:8-oxo-G mismatch. While MutYH very efficiently removed dA opposite 8-oxo-G, its activity on a rA:8-oxo-G mismatch was severely impaired with respect to the normal dA:8-oxo-G substrate (Fig. 4b; Supplementary Fig. 3B). Overall, these results indicate that incorporation of rNMPs opposite 8-oxo-G may impair repair of the damaged base by the BER pathway, potentially posing a threat to genomic stability.

### rCMP incorporation opposite 8-oxo-G in cell extracts

The data presented above, indicated that both Pols β and λ used rCTP as a substrate to bypass an 8-oxo-G lesion, generating a rC:8-oxo-G base pair, which was subsequently processed by hOGG1 with lowered efficiency. To better estimate to which extent Pols β and λ contribute to this potentially harmful event, we measured the incorporation opposite 8-oxo-G on a BER mimicking 1 nt-gap template, in extracts from mouse embryonic fibroblasts (MEFs) either proficient or deficient for Pol β or λ. Besides Pols β and λ, many other specialized Pols present in the cell, such as Pols μ, ν, η, ι, θ or κ, can efficiently fill 1 nt gaps. Like Pols β and λ, they are all resistant to the inhibitor aphidicolin, while replicative Pols α, δ and ɛ are sensitive to this inhibitor. The specialized Pol ζ is also aphidicolin sensitive, but its contribution to rNMPs incorporation in the cell was proposed to be minimal^38^. To determine the extent to which Pol β or λ contribute to potentially harmful rCMP incorporation opposite 8-oxo-G among specialized DNA repair Pols, aphidicolin was added to the reactions, thus blocking the incorporation by family B Pols α, δ, ɛ and ζ. The specific incorporation frequency (expressed as pmols of rCMP incorporated opposite 8-oxo-G per μg of extract) was calculated in each extract and normalized to the normal DNA synthesis (pmols of dCMP opposite guanine per μg of extract). As shown in Fig. 4c on the 1-nt gap on BER-mimicking template, Pol β^−/−^ MEFs displayed severely reduced rCMP incorporation opposite 8-oxo-G, with respect to Pol β^+/+^, as well as to Pol λ^+/+^ and Pol λ^−/−^ MEFs (compare lanes 1–3 with 4–12). In contrast, incorporation of dCMP opposite normal G was almost equal between Pol β^−/−^ and Pol β^+/+^ MEFs (Supplementary Fig. 3C, compare lanes 11 and 12 with lanes 14 and 15) and comparable with Pol λ extracts (Supplementary Fig. 3C,D). These data thus suggest that, among the aphidicolin-resistant Pols, Pol β seems to be responsible for most of the rCMP incorporation opposite 8-oxo-G in the extracts, while no statistically significant differences were found between Pol λ proficient and deficient cells (Fig. 4d).

## Discussion

Pols β and λ are the two major Pols involved in BER, one of the major DNA repair pathways operating in both resting and proliferating cells. In addition to providing the necessary gap-filling synthesis, we have previously shown that both Pols also operate in a specialized type of TLS in the context of MutYH-initiated repair of 8-oxo-G:A mismatches^25^. Since the contribution of BER to the incorporation of rNMPs into the genome is at present not fully understood, we decided to investigate the selectivity and fidelity of Pols β and λ with respect to rNMPs incorporation opposite normal and damaged bases under physiological concentrations of metal activators and nucleotides. Among the possible DNA damages, we selected 8-oxo-G, the very frequent oxidative stress DNA lesion, as a physiologically relevant example. Previous studies already revealed different sugar discrimination values among these Pols^18^, however, only with Mg^2+^ or with Mn^2+^ as the metal activator, as well as on different substrates. By comparing Pols β and λ under identical conditions (that is, in the presence of Mg^2+^ and on the same substrates), we also found clear differences in their sugar discrimination ability. Pol λ showed sugar selectivity of 5,100–7,500 and Pol β of 1,690–3,200 for incorporation of rCMP opposite a guanine, depending on the structure of the template (Table 1). These values were in agreement with previous studies, reporting rCTP/dCTP selectivity values of 4,000 and 2,000–8,000 for Pols λ and β, respectively^18^,^21^,^22^,^23^. While these findings suggest that Pols λ and β can incorporate rNMPs in normal DNA, currently little is known about their ability to introduce rNMPs when the template bears oxidative DNA damage. In a recent study, it was indicated that Pol ι and, to a much lesser extent, Pol η, perform TLS of 8-oxo-G damage by incorporating rNMPs^39^. Interestingly, in our study we found that the presence of an 8-oxo-G damage on the template strand significantly affected the Pol λ selectivity for rNTP/dNTP. On its preferred 1 nt-gap template, Pol λ showed a twofold increase in its preference for dCMP incorporation relative to rCMP incorporation opposite the lesion. Its fidelity was, on the other hand, unaffected since it preferred rCMP over rAMP to the same extent as dCMP over dAMP. We have shown previously that Pol λ is preferred over Pol β in the specialized MutYH-dependent BER of A:8-oxo-G mismatches^25^,^34^,^37^, due to its higher efficiency of incorporation opposite the lesion of the correct dCMP over the incorrect dAMP. The present data indicate that Pol λ will also minimize the chances of incorporating rNMPs opposite this lesion.

Pol β showed lower selectivity values than Pol λ for rCMP incorporation opposite an 8-oxo-G (Table 1). However, while Pol β can incorporate dAMP opposite 8-oxo-G almost as efficiently as dCMP^34^, when tested on the BER intermediate-mimicking 1 nt-gap template, it displayed a 13-fold preference for rCMP incorporation with respect to rAMP, compared with 1.6-fold preference for dCMP over dAMP on the same template (Table 1). In the crystal structure of the ternary complexes of Pol β with a (*syn*)8-oxo-G:(*anti*)dATP base pair^40^, the templating oxidized guanine was accommodated in the enzyme's active site in its *syn* conformation by a hydrogen bond between the 8-O of the guanine and the side chain of Arg283. The sugar-phosphate backbone around the lesion was also displaced of about 3.4 Å. The structure of a ternary complex of Pol β with an incoming rCTP and an undamaged template^41^, on the other hand, showed that a repulsive interaction between the backbone carbonyl of Tyr271 and the 2'OH group of the ribose counteracted base pairing of rCTP with the template G in the active site. In addition, rNTP binding disrupts a hydrogen bond between the hydroxyl group of Tyr271 and the primer end base, further disturbing the active site geometry. Thus, the combination of structural rearrangements required to accommodate both a templating 8-oxo-G in its *syn* conformation and an incoming rATP, may exacerbate the unfavourable interactions of Tyr271 with the ribose sugar and the primer end, thereby favouring the thermodynamically more stable rCTP pairing opposite 8-oxo-G. Crystal structures of Pol β in complex with an 8-oxo-G template and an incoming rNTP are needed to further clarify the molecular basis for this difference.

Experiments with extracts from cells defective in either Pol β or λ showed that, after inhibition of Pols α, δ, ɛ and ζ by aphidicolin, the majority of rCMP incorporation opposite 8-oxo-G was catalysed by Pol β. Interestingly, Pol λ seemed not to contribute significantly to rCMP-mediated bypass of 8-oxo-G. This is also in agreement with our kinetic data, showing that Pol λ discriminates against rCMP incorporation much more than Pol β (Table 1).

While the bulk of rNMPs incorporation in proliferating cells is due to DNA replication, our *in vitro* data suggest that also DNA synthesis during DNA repair on oxidative stress, among which the highly prominent is BER, may be a source of rNMPs incorporation.

On the basis of the selectivity values and on the known intracellular nucleotide concentrations^32^,^33^, we estimated the theoretical frequency of rCMP versus dCMP incorporation opposite a normal G or 8-oxo-G for three representative mammalian tissues. As shown in Table 2, Pol β may incorporate rCMP opposite a normal G with frequencies about twofold higher than Pol λ (0.38–5.8% versus 0.23–2.6%, respectively). These values are in agreement with the previously estimated average frequency of rNMPs incorporation by Pol β of 1.2% (ref. 23). In addition, under physiological rCTP/dCTP ratios, Pol β may incorporate rCMP opposite 8-oxo-G fourfold more frequently than Pol λ (Table 2). Our data show that Pol λ can bypass an 8-oxo-G with either rCMP or rAMP, depending on the template. However, its high discrimination against rNMPs incorporation and its preference for dCMP over dAMP, ensures that, even with strongly unbalanced rNTPs/dNTPs ratios, the vast majority of incorporation events will lead to dCMP incorporation (Table 2). Pol β, on the other hand, which is endowed with a lower sugar selectivity than Pol λ, during bypass of 8-oxo-G on a 1 nt-gap template, strongly discriminates against rAMP incorporation, thus ensuring that, at least, bypass will result in the incorporation of rCMP (wrong sugar/correct base) opposite the lesion, rather than rAMP (wrong sugar/wrong base). This distinction could become relevant, since we have shown here that the presence of rAMP opposite 8-oxo-G strongly inhibits the repair initiated by the MutYH glycosylase, while incorporation of rCMP would result in a 8-oxo-G:rC base pair, which can be still repaired by the OGG1 glycosylase, but at a reduced rate (Fig. 4a,b). During the final revision of the present manuscript, it has been reported that incorporation of rNMPs opposite 8-oxo-G in the context of CAG triplet repeats sequences, can negatively affect its subsequent repair, lending further support to our results^42^. Recent data also indicated that *Schizosaccharomyces pombe* RNaseH2 can remove rCMP incorporated opposite 8-oxo-G^43^, while *Escherichia coli* RNaseH2 in the presence of MutYH showed reduced efficiency in removing rAMP opposite 8-oxo-G^42^, suggesting that the ribonucleotide excision repair pathway can limit the deleterious effects of rNMPs accumulation during DNA repair, but possibly with some loss of efficiency. Thus, during evolution, it seems that specialized TLS Pols have been optimized to reduce the possible deleterious consequences of rNMPs incorporation opposite DNA lesions.

In summary, the data presented in this work support the notion that DNA repair can be a source of rNMPs accumulation, potentially leading to genomic instability. However, future work will be required to determine its precise impact to the cell. For example, accumulation of rNMPs in the brain, as well as the increase in the amount of oxidative DNA lesions, have been linked to neurodegeneration^44^,^45^. Given that Pol β is the most abundantly expressed Pol in adult neurons, where dNTPs pools are much lower than rNTPs and RNaseH2 is poorly expressed, its ability to incorporate rNMPs when copying both undamaged and damaged DNA, as shown in the present work, may contribute to these processes.

## Methods

### Chemicals

Deoxynucleotides were purchased from GeneSpin (Milan, Italy). Ribonucleotides were purchased by GE Healthcare (Uppsala, Sweden). The rNTPs stocks were double checked in house by HPLC for dNTPs contamination, which was found to be below the limit of detection (>99.6% purity). Labelled [γ-^32^P]ATP was purchased from Hartmann Analytic GmbH (Braunschweig, Germany). Adenosine, guanosine, cytidine and uridine standards were obtained from Sigma (St Louis, MO, USA). All the other reagents were of analytical grade and purchased from Fluka or Merck.

### Oligonucleotides

DNA oligonucleotides were synthesized by Purimex (Grebenstein, Germany) and purified from polyacrylamide denaturing gels. When indicated, oligonucleotides were 5′-labelled with T4 polynucleotide kinase (New England Biolabs) and [γ-^32^P]ATP, according to the manufacturer's protocol. Each labelled primer was mixed to the complementary template oligonucleotide at 1:1 (M/M) ratio in the presence of 25 mM Tris-HCl pH 8 and 50 mM KCl, heated at 75 °C for 10 min and then slowly cooled down at room temperature.

### Oligonucleotide sequences

40-mer template:

3′-ATAGGTGGTTATGATGGGATGCTATGATAGAGGTGAGTTG-5′.

The sequence underlined corresponds to the four templating bases at position +1, generated on annealing of the template to the 18merA, 19merT, 20merG and 21merC primers, respectively.

18merA primer:

5′-TATCCACCAATACTACCC-3′,

19merT primer:

5′-TATCCACCAATACTACCCT-3′,

20merG primer:

5′-TATCCACCAATACTACCCTA-3′,

21merC primer:

5′-TATCCACCAATACTACCCTAC-3′.

For the generation of the 1 nt-gap template, the 100mer was annealed to the 39-mer primer and to the 60-mer downstream oligo, which has been 5′-phosphorylated with unlabelled ATP by using T4 polynucleotide kinase (NEB).

100 mer template:

3′-ATGTTGGTTCTCGTATGCTGCCGGTCACG GCTTAAGTGTXCCACAACACACAACCAACACCACCACAACACACCAACAACCACAACACACACAACCAC AC-5′,

where X=G or 8-oxo-G. The sequence underlined is the one complementary to the 39-mer primer.

39-mer primer:

5′-TACAACCAAGAGCATACGACGGCCAGTGCCGAATTCACA-3′

60-mer downstream oligonucleotide:

5′-GGTGTTGTGTGTTGGTTGTGGTGGTGTTGTGTGGTTGTTGGTGTTGTGTGTGTTGGTGTG-3′.

### Enzymes and proteins

Human recombinant Pols λ and β (ref. 34) and human recombinant glycosylases MutYH and hOGG1 (refs 37, 46), were used.

### Cell lines and culturing conditions

Immortalized Pol λ^+/+^, Pol λ^−/−^, Pol β^+/+^ and Pol β^−/−^ MEFs^47^,^48^ were grown in a humidified 5% CO_2_ atmosphere in Dulbecco's modified Eagle's medium containing GlutaMAX-I supplemented with 10% fetal bovine serum and 100 U ml^−1^ penicillin–streptomycin (all obtained from Gibco, Invitrogen).

### DNA polymerase assays

All reactions were done in a final volume of 10 μl and incubated for 10 min at 37 °C in the presence of 5′-labelled p/t at the concentrations stated in the figure legends. Pol λ, β reaction buffer: 50 mM Tris pH 7.5, 1 mM DTT, 0.2 mg ml^−1^ BSA. Mg^++^ was included for all Pols at 5 mM, unless otherwise stated in the figures or figure legends. When crude extracts were used, the reaction mixture was supplemented with 0.1 mg ml^−1^ aphidicolin and 1 μM of a 49mer single-stranded DNA oligonucleotide with a non-complementary sequence to the DNA substrate, to inhibit exonuclease degradation. Crude whole-cell extracts were prepared as described in ref. 49. Proteins, Mg^2+^, Mn^2+^ and nucleotides were in the concentration specified in the figures and figure legends. For denaturing gel analysis of the DNA products, the reaction mixtures were stopped by addition of standard denaturing gel loading buffer (95% formamide, 10 mM EDTA, xylene cyanol and bromophenol blue), heated at 95 °C for 5 min and loaded on a 7-M urea 12% polyacrylamide (PA) gel. The reaction products were analysed by using Molecular Dynamics Phosphoimager (Typhoon Trio, GE Healthcare) and quantified by the program Image Quant.

### DNA glycosylase assays

*hOGG1* was incubated with 5′-labelled ds 100-mer DNA with 8-oxo-G:dC or 8-oxo-G:rC mispairs. On incubation, the reaction products were treated with NaOH (2M), heated for 15 min at 75 °C and reactions analysed on a 7-M urea 10% polyacrylamide gel. The reactions were performed at 37 °C in final volume of 10 μl containing 20 fmol 5′-labelled ds 100-mer DNA with 8-oxo-G:dC or 8-oxo-G:rC, 20 mM Tris-HCl (pH 8), 1 mM DTT, 1 mM EDTA and 0.1 mg ml^−1^ BSA. hOGG1 concentration and incubation times varied and are indicated in figures. On incubation hOGG1 reaction products were treated with 1 μl NaOH (2 M), heated for 15 min at 75 °C and reactions stopped by the addition of denaturing gel loading buffer and heating at 95 °C for 5 min. The samples were separated on 7 M urea 10% PA gel, analysed by Phosphoimager and quantified by GelEval 1.35 scientific imaging software (FrogDance Software, UK).

*MutYH* assay^37^ was performed in 10 μl reactions containing 25 mM Hepes–KOH pH 6.8, 5 mM EDTA, 1.5% glycerol, 50 μM ZnCl_2_, 50 mM NaCl, 7.5 mM MgCl_2_, 20 fmol 5′-labelled ds 100-mer DNA with dA:8-oxo-G or rA:8-oxo-G, 200 nM MutYH and varying amounts of APE1 as indicated in Supplementary Fig. 3. Reactions were stopped by the addition of denaturing gel-loading buffer, heating at 95 °C for 5 min and separated on 7 M urea 10% PA gel. The products were detected by Phosphoimager and quantified by GelEval 1.35 scientific imaging software (FrogDance Software, UK).

### Kinetic analysis

Due to the highly distributive nature of the reaction and the relatively low efficiency of rNTPs incorporation, the enzymes and the DNA substrate were used at similar concentrations. This implies that, at equilibrium, the concentration of the binary enzyme–DNA complex does not approximate the total enzyme concentration, but there is always a fraction of enzyme not bound to the DNA substrate, that does not participate in catalysis. Thus, to account for the fraction of enzyme not bound to the DNA substrate at equilibrium, the variation of the nucleotide incorporation rates (*v*) as a function of the nucleotide substrate concentration was fitted to the modified Briggs–Haldane equation:


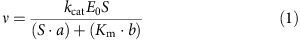


where *k*_cat_ is the apparent catalytic rate, *E*_0_ is the input enzyme concentration, *S* is the variable nucleotide substrate concentration, *K*_m_ is the apparent equilibrium dissociation constant of the nucleotide substrate from the catalytically active ternary complex, *a* and *b* are two constants, whose values were kept fixed during the computer fitting and were calculated from the following expressions:


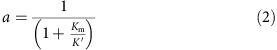



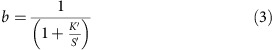


where *K*_m_ is the same as in equation (1), *K*′ is the apparent dissociation constant for binding to the DNA substrate and *S'* is the concentration of DNA use in each experiment. The values of *K′* used for the fitting process were for Pol β 22 nM (ref. 50) and for Pol λ 29 nM (ref. 51).

Under conditions of forced termination (single nucleotide incorporation), *K*_m_=*K*_s_(*k*_off_/(*k*_pol_+*k*_off_)) and *k*_cat_=*k*_pol_(*k*_off_/(*k*_pol_+*k*_off_)). Where *K*_s_ is the Michelis constant for nucleotide binding, *k*_off_ is the dissociation rate of the enzyme from the DNA substrate and *k*_pol_ is the polymerization rate. Hence *k*_cat_/*K*_m_=*k*_pol_/*K*_s_.

Nucleotide concentrations used were in the 0.005–5 μM range for dNTPs and 0.5–2,000 μM range for rNTPs.

Time-course experiments were fitted to the exponential equation:


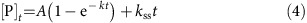


where [P]_*t*_ is the product concentrations at any given time point, *t* is time, *k* is the rate for the exponential phase, *k*_ss_ is the rate of the linear phase and *A* is a constant.

Fitting was obtained with the GraphPad Prism 3.0 computer program.

### Electronic image manipulation

Linear transformations have been applied in some instance to the whole images using the exposure/brightness filters of Adobe Photoshop CS6 with the sole purpose of reducing excessive background. No masking/enhancement was applied to any specific feature of the images.

## Additional information

**How to cite this article**: Crespan, E. *et al.* Impact of ribonucleotide incorporation by DNA polymerases β and λ on oxidative base excision repair. *Nat. Commun.* 7:10805 doi: 10.1038/ncomms10805 (2016).

## Supplementary Material

Supplementary Information Supplementary Figures 1-3

## Figures and Tables

**Table 1:** Kinetic parameters for rNMPs incorporation by DNA polymerases **λ** and **β**.

	***K***_m_* **(μM)**	***k***_cat_* **(min**^−**1**^**)**	***k***_**cat**_**/*****K***_m_* **(M**^**−1**^** s**^**−1**^**)**	***f***_**inc**_† **dNTP/rNTP**	***f***_**inc**_ **rNTP/dNTP**	***f***_**inc**_ **C** **versus** **A**
*1 nt-gap control*						
** **Pol λ						
** **rCTP	83±4	0.024±0.003	4.8		1.3 × 10^−4^	NA
** **dCTP	0.018±0.004	0.039±0.002	3.6 × 10^4^	7,500		NA
** **Pol β						
** **rCTP	47±3	0.034±0.002	12		2.9 × 10^−4^	NA
** **dCTP	0.03±0.004	0.074±0.002	4.1 × 10^4^	3,400		NA
						
*1 nt-gap 8-oxo-G*						
Pol λ						
** **rCTP	50±10	0.06±0.003	20		7.1 × 10^−5^	5.5
** **dCTP	0.007±0.001	0.12±0.01	28 × 10^4^	14,000		4.5
** **rATP	185±20	0.04±0.01	3.6		5.8 × 10^−5^	
** **dATP	0.015±0.001	0.056±0.01	6.2 × 10^4^	17,200		
** **Pol β						
** **rCTP	29±1	0.018±0.002	10.3		3.1 × 10^−4^	13.4
** **dCTP	0.01±0.01	0.02±0.001	3.3 × 10^4^	3,200		1.6
** **rATP	367±30	0.017±0.003	0.77		3.9 × 10^−5^	
** **dATP	0.017±0.002	0.021±0.002	2 × 10^4^	25,600		
*39/100mer control*						
** **Pol λ						
** **rCTP	38±0.5	0.008±0.0003	3.5		1.9 × 10^−4^	NA
** **dCTP	0.03±0.005	0.032±0.007	18 × 10^3^	5,140		NA
** **Pol β						
** **rCTP	64±7	0.015±0.003	3.9		6 × 10^−4^	
** **dCTP	0.05±0.01	0.02±0.002	6.6 × 10^3^	1,690		
*39/100mer 8-oxo-G*						
** **Pol λ						
** **rCTP	155±18	0.02±0.004	2.1		0.7 × 10^−4^	0.9
** **dCTP	0.05±0.004	0.08±0.008	27 × 10^3^	12,800		4
** **rATP	200±5	0.027±0.009	2.2		3.3 × 10^−4^	
** **dATP	0.15±0.004	0.06±0.01	6.6 × 10^3^	3,000		
** **Pol β						
** **rCTP	73±13	0.007±0.002	1.6		2.1 × 10^−4^	2
** **dCTP	0.04±0.01	0.018±0.002	7.5 × 10^3^	4,680		3
** **rATP	150±15	0.007±0.003	0.8		5.7 × 10^−4^	
** **dATP	0.15±0.02	0.021±0.003	2.3 × 10^3^	2,800		

^*^The meaning of the kinetic parameters *K*_m_, *k*_cat_ and *k*_cat_/*K*_m_ and their calculations are described in the Methods section. Values are the means of two independent estimates ±s.d.

^†^*f*_inc_, relative incorporation frequencies for the different nucleotide pairs, defined as the ratio of the respective *k*_cat_/*K*_m_ values.

**Table 2:** Estimated misincorporation frequencies of rCTP versus dCTPs opposite 8-oxo-G or a normal G, for DNA polymerases **β** and **λ** under physiological dNTPs/rNTPs concentrations.

**Tissue**	**rCTP/dCTP ratio**	**Template G**	**Template 8-oxo-G**
		***f***(rCTP/dCTP)* × 10^**−3**^	***f***(rCTP/dCTP)* × 10^**−3**^
*Pol λ*			
Skin fibroblasts†	18†	2.3	1.3
Lymphocytes‡	200‡	26	14
Liver‡	13‡	1.7	0.9
*Pol β*			
Skin fibroblasts	18†	5.2	5.6
Lymphocytes	200‡	58	62
Liver	13‡	3.8	4

^*^Frequencies of incorporation for each ribo-/deoxy-nucleotide substrate pair were calculated according to the Cornish–Bowden relationship: *f*(rCTP/dCTP)=(*k*_cat_/*K*_m_rCTP)/(*k*_cat_/*K*_m_dCTP)·(rCTP)/(dCTP) using the values from Table 1 for the 1-nt gap and from refs 32, 33.

^†^The rCTP and dCTP concentrations have been taken from ref. 33.

^‡^The rCTP and dCTP concentrations have been taken from ref. 32.
