# Supplementary material for: Impact of ribonucleotide incorporation by DNA polymerases β and λ on oxidative base excision repair
Source: Nat Commun. 2016 Feb 26;7:10805. doi: 10.1038/ncomms10805 (PMC4773436; doi:10.1038/ncomms10805)
Supplement: Supplementary Information — Supplementary Figures 1-3 [file ncomms10805-s1.pdf]

Supplementary Figure 1

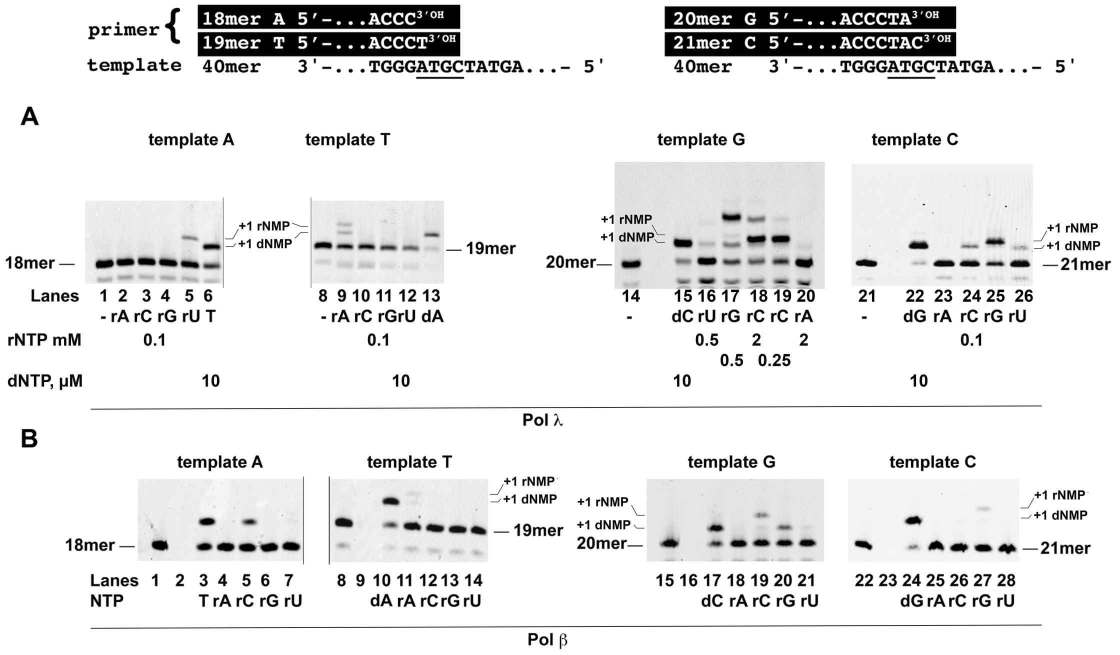

Suppl. Figure 1. A. Pol λ (20 nM) was incubated in the presence of 10 nM of the 5'labelled 18A/40mer p/t (lanes 1-6), 19T/40mer p/t (lanes 8-13), 20G/40mer p/t (lanes 14-20), 21C/40mer p/t (lanes 21-26), in the presence of different combinations of rNTPs and dNTPs, as indicated. Lanes 1, 8, 14, 21, control reactions in the absence of nucleotides. **B**. As in panel A, but in the presence of 50 nM Pol β and 100 nM templates. Lanes 1, 8, 15, 22, control reactions in the absence of nucleotides. Solid lines indicate different parts of the same gel.

Supplementary Figure 2

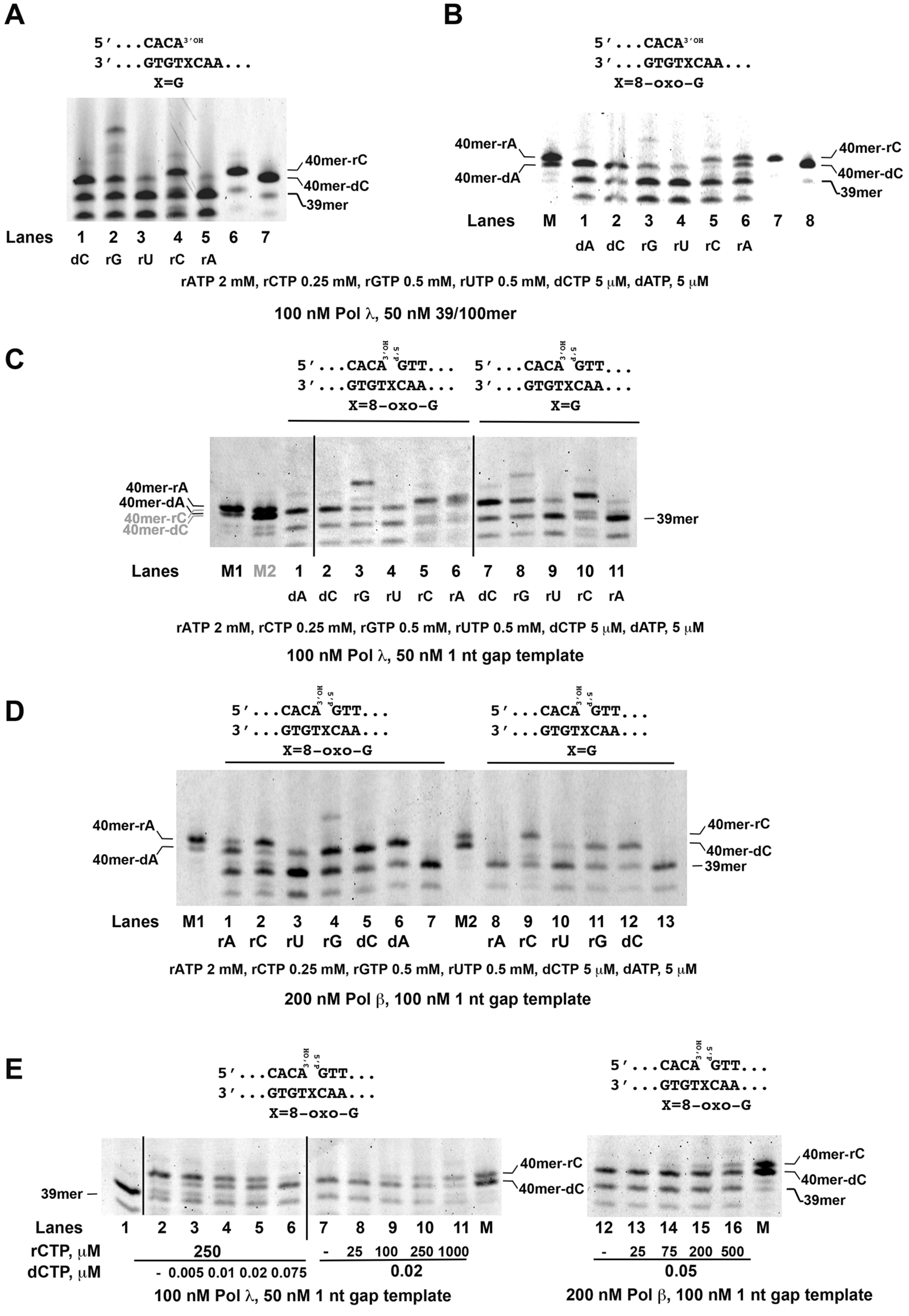

Suppl. Figure 2. Enzyme, substrate and nucleotide concentrations are indicated at the bottom of each panel. Solid lines indicate that different portions of the

same gel were brought next to each other for better clarity. **A** Pol λ activity was measured on the undamaged 5'-labelled 39/100mer p/t, in the presence of each of the four rNTPs. Lane 6, 40mer rCMP-terminated oligonucleotide marker. Lane 7, dCMP-terminated 40mer oligonucleotide marker. **B**. As in panel A, but in the presence of the 5'-labelled 39/100mer p/t containing 8-oxo-G. Lane M, a mixture of 5'-labelled 40mer oligonucleotides bearing either dCMP or rCMP as the terminal nucleotide, as markers. **C**. Pol λ activity was tested in the presence of the 5'-labelled 39/60/100mer 1nt gap template either bearing 8-oxo-G (lanes 1 – 6) or undamaged (lanes 7 – 11), in the presence of each of the four rNTPs. Lane M1, a mixture of 5'-labelled 40mer oligonucleotides bearing either dAMP or rAMP as the terminal nucleotide, as markers. Lane M2, a mixture of 5'-labelled 40mer oligonucleotides bearing either dCMP or rCMP as the terminal nucleotide, as markers. **D**. As in panel C, but in the presence of Pol β. Lane 13, control reaction in the absence of nucleotides. **E**. Pol λ (lanes 2 – 11) or Pol β (lanes 12 – 16) activity was tested in the presence of fixed concentrations of rCTP (lanes 2 – 6) or dCTP (lanes (7 – 16), alone (lanes 2, 7, 12) or in combination with increasing concentrations of rCTP (lanes 8 -11, 13 – 16) or dCTP (lanes 3 – 6). Lanes M, a mixture of 5'-labelled 40mer oligonucleotides bearing either dCMP or rCMP as the terminal nucleotide, as markers. Lane 1, 39mer primer alone.

Supplementary Figure 3

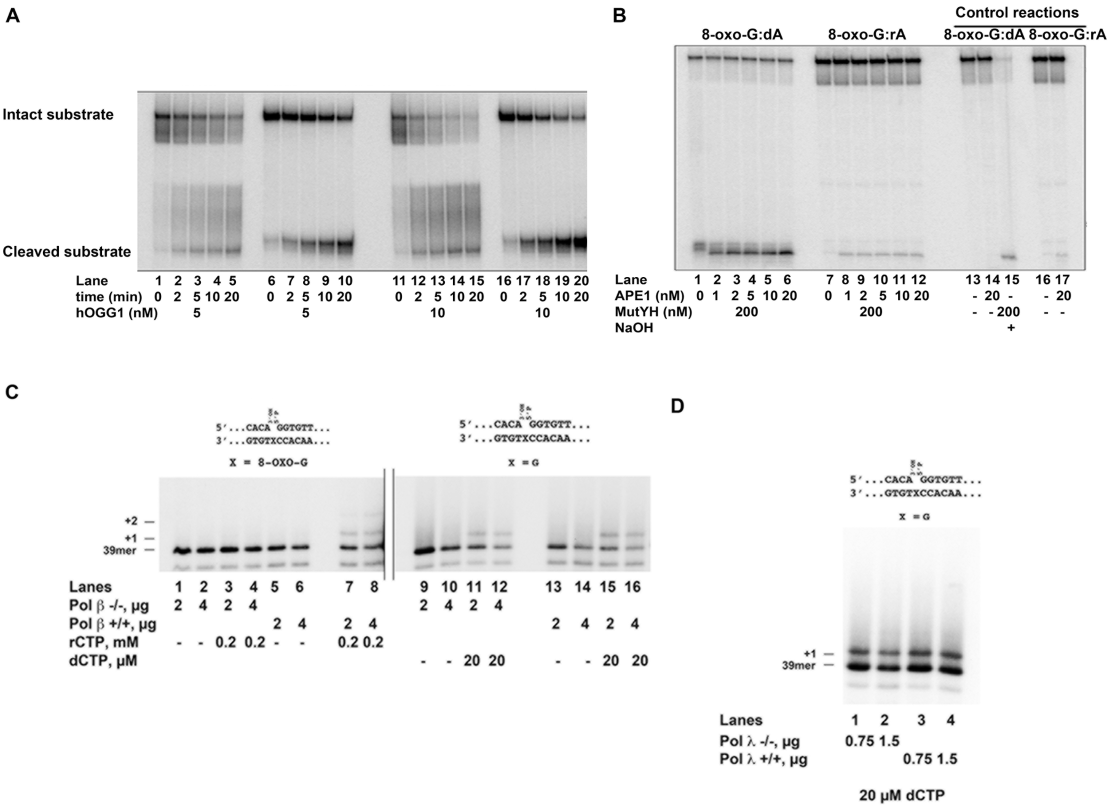

Suppl. Figure 3. A. Time course of the reaction catalyzed by hOGG1 at 5 nM (lanes 1-10) or 10 nM (lanes 11-20) on the ds100mer substrate containing a 8-oxo-G:dC (lanes 1-5; 11-15) or a 8-oxo-G:rC (lanes 6-10; 16-20) mispair. **B.** Titration of APE1 in the presence of MutYH on the ds100mer substrate containing a 8-oxo-G:dA (lanes 1-6) or a 8-oxo-G:rA (lanes 7-12) mispair. Lanes 13-17, various control reactions for the 8-oxo-G:dA (lanes 13-15) and 8-oxo-G:rA (lanes 16-17) templates. The conditions are specified at the bottom of each lane. **C**. Increasing amounts of extracts from Pol $\beta^{-/-}$ (lanes 1 - 4; 9 - 12) or Pol $\beta^{+/+}$ (lanes 2 - 8; 13 - 16) cells, were titrated with the 1 nt gap template containing an 8-oxo-G (lanes 1 - 8) or undamaged (lanes 9 - 16), in the absence (lanes 1, 2, 5, 6, 9, 10, 13, 14) or in the presence of rCTP (lanes 3, 4, 7, 8) or dCTP (lanes 11, 12, 15, 16). Solid lines indicate that lanes 1 - 8 and 9 - 16 represent different portions of the same gel. **D**. Increasing amounts of extracts from Pol $\lambda^{-/-}$ (lanes 1,

2) or Pol λ $^{+/+}$ (3, 4) cells were titrated with the undamaged 1 nt gap template in the presence of dCTP.
